# Reduced Total Subgingival Bacterial Load in Patients with Ischaemic Stroke: A Case–Control Study of the Subgingival Microbiome and Carotid Atherosclerosis

**DOI:** 10.3390/jcm15176703

**Published:** 2026-08-29

**Authors:** Anita Siewko, Szymon J. Jurga, Maksymilian Aleksander Brzezicki, Izabela Wojtasz, Anita Nowak, Filip Czop, Maja Jurga, Iwona Towpik, Lukasz Dzieciuchowicz, Jaroslaw Piskorski, Radoslaw Kazmierski

**Affiliations:** 1 Department of Neurology, Institute of Medical Sciences, University of Zielona Góra, Zyty 28, 65-046 Zielona Góra, Poland; a.siewko@szpital.zgora.pl (A.S.); s.jurga@inm.uz.zgora.pl (S.J.J.); i.wojtasz@inm.uz.zgora.pl (I.W.); r.kazmierski@inm.uz.zgora.pl (R.K.); 2 Department of Neural Engineering and Space Medicine, Institute of Medical Sciences, University of Zielona Góra, Zyty 28, 65-046 Zielona Góra, Poland; f.czop@szpital.zgora.pl; 3 Jesus College, University of Oxford, Turl Street, Oxford OX1 3DW, UK; 4 Department of Vascular Surgery and Vascular Disease, Institute of Medical Sciences, University of Zielona Góra, Zyty 28, 65-046 Zielona Góra, Poland; a.nowak@szpital.zgora.pl (A.N.); l.dzieciuchowicz@inm.uz.zgora.pl (L.D.); 5 Faculty of Medicine and Dentistry, Medical University of Warsaw, Żwirki i Wigury 61, 02-091 Warsaw, Poland; s090147@student.wum.edu.pl; 6 Department of Internal Medicine, Institute of Medical Sciences, University of Zielona Góra, Zyty 28, 65-046 Zielona Góra, Poland; i.towpik@inm.uz.zgora.pl; 7 Institute of Physics, University of Zielona Góra, Prof. Z. Szafrana 4a, 65-516 Zielona Góra, Poland; j.piskorski@if.uz.zgora.pl

**Keywords:** stroke, periodontitis, oral microbiome, bacterial load, carotid intima-media thickness, case–control study

## Abstract

**Background/Objectives**: Periodontal infection has been associated with ischaemic stroke, usually as a matter of burden: more pathogens, more vascular risk. That framing derives from stroke-free populations and rests largely on surrogate endpoints, chiefly carotid intima-media thickness (IMT). Whether it holds once stroke has occurred is unclear. **Methods**: We concurrently recruited 50 patients with ischaemic stroke and 48 stroke-free controls in western Poland. The subgingival microbiome was quantified for nine periodontal taxa and the total bacterial count, analysed on the log10 scale, with carotid IMT and standard biochemistry recorded in parallel. Associations with stroke were assessed by univariable and multivariable logistic regression with backward elimination, validated by optimism-corrected bootstrap. **Results:** Stroke patients had fewer teeth, deeper pockets, poorer oral hygiene, higher carotid IMT, white cell count and C-reactive protein (all *p* < 0.001). *Treponema denticola* and *Tannerella forsythia* were higher in cases and *Fusobacterium nucleatum* lower. Total bacterial load was lower in cases (mean 9.3, SD 0.9 versus 10.2, SD 0.9 log10, *p* < 0.001) and remained independently and inversely associated with stroke after adjustment (adjusted odds ratio 0.09 per ten-fold increase; 95% CI 0.02–0.38). This was not explained by tooth loss: load was uncorrelated with tooth number (rho 0.12, *p* = 0.26) and the association persisted after normalisation per tooth. Discrimination was high (c-statistic 0.970; optimism-corrected 0.940). **Conclusions**: The subgingival microbiome shifted with stroke in a taxon-specific manner, yet total bacterial load was lower, not higher, in stroke patients. Because microbial and inflammatory markers were measured after the event, these associations most plausibly reflect peri-stroke change and case–control structure rather than a forward-causal signal, marking a boundary on the periodontal-burden paradigm.

## 1. Introduction

Periodontal infection is a common, treatable bacterial disease of the tooth-supporting tissues that has been associated with ischaemic stroke. The relationship is usually framed as a matter of burden, more pathogens meaning more vascular risk, though that framing derives from stroke-free populations. Periodontitis arises from a dysbiotic shift in the subgingival biofilm rather than from any single organism [1]. Within this altered community, three Gram-negative anaerobes, *Porphyromonas gingivalis*, *Tannerella forsythia* and *Treponema denticola*, constitute the red complex and correlate most closely with clinical attachment loss and pocket depth [2]. *Porphyromonas gingivalis* acts as a keystone pathogen: even at low abundance it reshapes the surrounding microbiome and subverts the host response, amplifying inflammation out of proportion to its numbers [1,3]. Community composition and total bacterial abundance are therefore distinct quantities that need not move together, a distinction central to the present study.

Several non-exclusive pathways could underlie such a relationship. Transient bacteraemia during chewing and toothbrushing, accentuated by periodontal inflammation, delivers viable organisms and their products into the circulation [2]. *Porphyromonas gingivalis* invades vascular and coronary endothelium, upregulates adhesion molecules and chemokines, recruits monocytes, promotes foam-cell formation and impairs endothelial repair, while its gingipains degrade junctional proteins and its lipopolysaccharide activates platelets and induces procoagulant activity [4]. Systemically, chronic periodontal inflammation sustains the raised C-reactive protein and interleukin-6 that accompany atherosclerosis [3], and oral inoculation with *Porphyromonas gingivalis* accelerates plaque accumulation in animal models [5].

These mechanisms are matched by consistent epidemiology, which has almost uniformly pointed in one direction: more periodontal disease, and more pathogen burden, accompany more cerebrovascular disease. Severe periodontitis is an independent risk factor for cerebral ischaemia, conferring a more than fourfold excess in younger men [6], and a meta-analysis of 50 case–control studies placed the odds ratio for chronic bacterial infection, principally *Chlamydia pneumoniae*, *Helicobacter pylori* and *Mycobacterium tuberculosis* rather than periodontal pathogens, at 1.70 (95% CI 1.57–1.84) [7]. The mechanistic bridge invoked is subclinical atherosclerosis: severe periodontitis is associated with greater carotid intima-media thickness (IMT) [8], a higher subgingival pathogen burden tracks with greater IMT [9], and systemic exposure to *Porphyromonas gingivalis* predicts incident stroke [10]. Notably, each of these microbiological analyses was performed in people free of prior stroke, and so describes an antecedent, forward-directed relationship in which greater burden precedes greater disease.

Sequencing has since reframed the question from how much to which. Saliva studies in stroke report altered composition rather than uniform overgrowth, implicating genera such as *Streptococcus* spp., *Prevotella* spp., *Veillonella* spp. and *Fusobacterium* spp. [11,12], and oral bacterial DNA recovered from cerebral thrombi is dominated by *Streptococcus* spp. and *Prevotella* spp., with classical periodontal pathogens largely absent [13]; viridans streptococci have since been demonstrated within thrombus aspirates and within ruptured carotid atheroma by bacterial immunohistochemistry [14]. The microbial signal in manifest stroke may therefore be qualitative and directional rather than a simple rise in load. Yet total load and individual-pathogen abundance have seldom been measured together in the same patients, so their relationship in manifest stroke is largely uncharacterised, and the burden-based evidence rests on surrogate endpoints in stroke-free cohorts. Studies of stroke patients are typically small, based on a single sequenced specimen, and lacking a contemporaneous control group, leaving unresolved whether any association reflects antecedent exposure or peri-event change.

We therefore quantified nine named periodontal pathogens by a targeted method, together with the total bacterial count, in patients with ischaemic stroke and in concurrently recruited stroke-free controls, with carotid IMT measured in parallel. Our aim was to characterise how oral microbial burden and composition relate to manifest ischaemic stroke, and to test whether the forward-directed, burden-based paradigm derived from stroke-free cohorts holds in patients who have already had a stroke.

## 2. Materials and Methods

### 2.1. Study Design and Population

This observational case–control study was conducted between 2020 and 2023 at a tertiary referral teaching hospital in western Poland. Recruitment was concurrent and open to both arms, meaning cases and controls were enrolled in parallel over the same period and assessed with an identical protocol, reducing temporal drift between groups. We enrolled 50 patients with ischaemic stroke (cases) and 48 stroke-free controls (98 analysed). Ischaemic stroke was defined as an episode of sudden focal neurological deficit persisting beyond 24 h, with an infarct in the corresponding vascular territory on computed tomography or magnetic resonance imaging. The diagnosis was made by a neurologist during hospital admission and no case was based on self-report. Transient ischaemic attack was an exclusion criterion, so all cases were cerebral infarcts [15]. Eligibility for both arms required age 40 years or older and at least three natural teeth to permit subgingival sampling; controls additionally had no history of cardiovascular disease. Controls were volunteers recruited at the hospital by public announcement. There was no further screening beyond the assessments described below, which were identical in both groups. Participants were not individually matched, although the groups proved to be of similar age. Controls were not selected for the absence of other conditions, and a small number had minor carotid stenosis on duplex. Stroke severity was recorded as the National Institutes of Health Stroke Scale (NIHSS) score on admission and at discharge, and functional status as the modified Rankin Scale (mRS) score at the same two time points.

### 2.2. Ethics

The study was conducted in accordance with the Declaration of Helsinki and approved by the Regional Ethics Committee (approval no. 15/151/2021). Written informed consent was obtained from all participants, or from a legal representative where a participant was unable to consent.

### 2.3. Clinical and Laboratory Assessment

Recorded variables comprised age, sex and smoking status; dental status (number of teeth, periodontal pocket depth, dental calculus, oral hygiene category); a full lipid panel, glucose, C-reactive protein (CRP), a complete blood count including the white cell count (WBC), creatinine and estimated glomerular filtration rate. Oral hygiene was graded by the examining clinician on visual inspection of supragingival plaque and calculus as good (no or minimal visible plaque, confined to isolated surfaces), medium (visible plaque on multiple surfaces without generalised deposits) or poor (generalised visible plaque with abundant calculus); no formal plaque index was applied. Periodontal pocket depth was measured with a manual periodontal probe, the model of which was not recorded, at one site per tooth and recorded to the nearest 1 mm; the value analysed is the mean pocket depth across the teeth examined. Carotid arteries were assessed by duplex Doppler ultrasonography on a GE LOGIQ system (GE Healthcare, Chicago, IL, USA) using a 4–13 MHz linear-array transducer for IMT, plaque and stenosis. In line with the Mannheim consensus [16], IMT was measured automatically 1 cm proximal to the carotid bulb, and plaque was defined as focal thickening 1.5 mm or more, or 50% or more relative to surrounding IMT. Left ventricular ejection fraction and the presence of atrial fibrillation were taken from the routine clinical record; transthoracic echocardiography and ambulatory electrocardiography were performed as part of standard care rather than to a protocol specified for this study.

### 2.4. Microbiological Assessment

The subgingival microbiome was quantified for nine periodontal taxa (*Porphyromonas gingivalis*, *Treponema denticola*, *Tannerella forsythia*, *Prevotella intermedia*, *Peptostreptococcus* (*Micromonas*) *micros*, *Fusobacterium nucleatum*, *Capnocytophaga gingivalis*, *Aggregatibacter actinomycetemcomitans* and *Eubacterium nodatum*), with the total bacterial count determined in parallel. Counts are right-skewed and were analysed on the log10 scale; univariable taxon models used log10(count + 1) to keep zero counts defined. Subgingival samples were analysed with the PET plus assay (Zestaw diagnostyczny PET; MIP Pharma GmbH, Blieskastel, Germany, distributed in Poland by MIP Pharma Polska, Gdansk), a commercial molecular test for pathogens causing periodontitis and peri-implantitis. The nine taxa and the total bacterial count were determined qualitatively and quantitatively by real-time polymerase chain reaction. For each participant, a single pooled subgingival sample was analysed, taken from two subgingival sites selected as the deepest periodontal pocket available in each of two teeth, using one sterile paper point per site, each left in the pocket for 10 s and both transferred to the same transport tube, giving a single pooled specimen. Sampling was performed on the first day of admission, during the day. Results are reported by the laboratory as the absolute number of organisms in the pooled sample and as the percentage of the total bacterial count, and organisms below the detection threshold are reported as not detected; these were entered as zero for analysis. The total bacterial count was determined with universal bacterial primers rather than by summation of the individual taxa. DNA extraction and amplification were performed by the laboratory according to the standard workflow for the assay, and the laboratory was blinded to whether a sample came from a case or a control. The detection threshold, the calibration procedure and the number of technical replicates and internal run controls are proprietary to the assay and are not disclosed by the manufacturer, and could not therefore be reported here. Samples were posted to the laboratory immediately after collection in the transport vials supplied with the assay, and were analysed on the day of receipt. Samples for this assay are processed at the manufacturer’s laboratory in Germany.

### 2.5. Statistical Analysis

Continuous variables were summarised as mean (SD) when normally distributed (Shapiro–Wilk *p* > 0.05 in both groups) and otherwise as median [interquartile range], and compared with the Student *t*-test or Mann–Whitney U test. Categorical variables were summarised as *n* (%) and compared with the chi-squared or Fisher exact test. Associations with stroke were assessed by univariable logistic regression and by a multivariable model derived from a pre-specified candidate set (age, sex, number of teeth, carotid IMT, WBC, total bacterial count) by backward elimination. Odds ratios (OR) are reported with 95% Wald confidence intervals; the OR for total bacterial count is expressed per ten-fold increase and that for IMT per 0.1 mm. Discrimination was quantified by the area under the receiver-operating-characteristic curve, with an optimism-corrected bootstrap (500 resamples repeating the full selection). Calibration was assessed by the Hosmer–Lemeshow test, fit by McFadden and Nagelkerke pseudo-R^2^, and collinearity by variance-inflation factors. Confounding by tooth loss was examined by correlating load with tooth number, then reanalysing load normalised per tooth and with tooth number as a covariate. A two-sided *p* < 0.05 was significant. Analyses used R 4.5.2 (R Foundation for Statistical Computing, Vienna, Austria). Associations between stroke severity and the oral and microbiological variables were assessed with Spearman rank correlation.

## 3. Results

### 3.1. Baseline Characteristics

**Figure 1:**
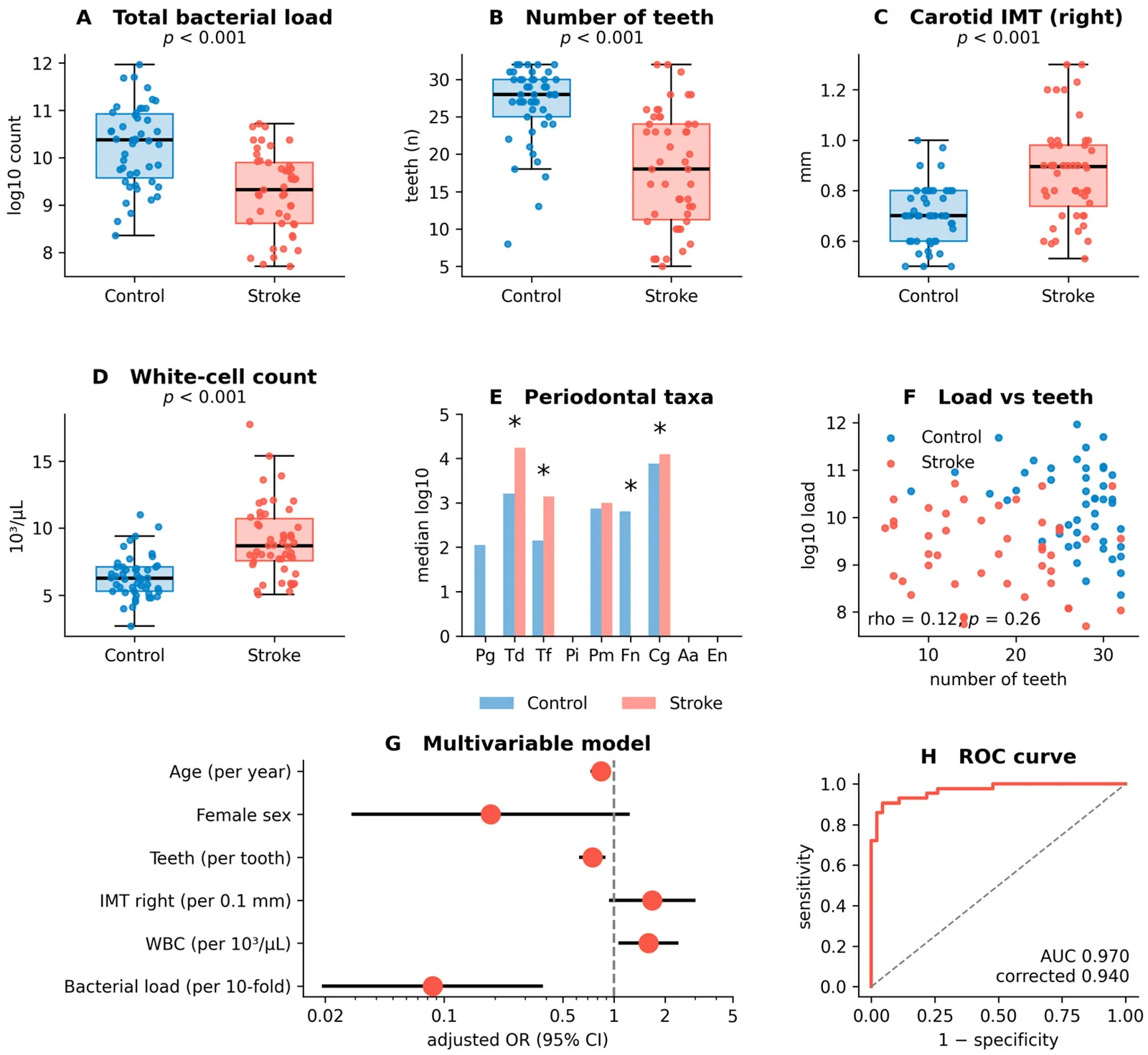
Consolidated summary of study findings. (**A**–**D**) Group comparisons for total bacterial load, number of teeth, right carotid intima-media thickness and white cell count; boxes show median and interquartile range, points show individual participants. (**E**) Median log10 abundance of all nine periodontal taxa by group, in the order given in the Methods; asterisks mark taxa differing at *p* < 0.05 (Td, Tf, Fn, Cg). (**F**) Total bacterial load versus number of teeth, showing absence of correlation (Spearman rho 0.12, *p* = 0.26). (**G**) Adjusted odds ratios (95% CI) from the final model on a log scale; points left of 1.0 favour control (lower odds of stroke) and points right favour stroke. (**H**) Receiver-operating-characteristic curve for the final model (apparent AUC 0.970; optimism-corrected 0.940).

The groups were of similar age (66.0 vs. 67.4 y; *p* = 0.377); men predominated among cases (74.0% vs. 43.8%; *p* = 0.005). Cases had fewer teeth (median 18 vs. 28), deeper pockets, and poorer oral hygiene (poor hygiene 40.0% vs. 12.5%; all *p* < 0.001). Carotid IMT was higher bilaterally, and WBC (8.7 vs. 6.2 × 10^3^/µL), CRP (3.2 vs. 0.1 mg/dL) and glucose were higher in cases, while total, HDL and LDL cholesterol and ejection fraction were lower (Table 1; Figure 1A–D). Atrial fibrillation was uncommon in both groups and was documented in 2 of 47 cases and 1 of 48 controls (*p* = 0.617). Among the 50 cases, the median NIHSS score was 5 [3–8] on admission and 2 [1–4] at discharge, and the median mRS score was 0 [0–1] on admission and 1 [0–2] at discharge. Neuroimaging showed a territorial infarct in 40 patients and a lacunar infarct in 6; in the remaining 4, the initial computed tomography was unremarkable and the infarct was confirmed on magnetic resonance imaging. Fifteen patients received intravenous thrombolysis and 4 underwent mechanical thrombectomy, and 7 had a previous stroke.

### 3.2. Subgingival Microbiome

Total bacterial load was lower in cases than controls (mean 9.3 vs. 10.2 log10; *p* < 0.001; Table 2; Figure 1A). *Treponema denticola* (4.2 vs. 3.2; *p* = 0.013) and *Tannerella forsythia* (3.1 vs. 2.1; *p* = 0.047) were higher in cases, and *Capnocytophaga gingivalis* marginally so (*p* = 0.049), whereas *Fusobacterium nucleatum* was lower (*p* = 0.020). *Porphyromonas gingivalis*, *Prevotella intermedia*, *Peptostreptococcus micros*, *Aggregatibacter actinomycetemcomitans* and *Eubacterium nodatum* did not differ (Figure 1E). On the log10 scale, values in controls versus stroke patients were: total bacterial load (mean, SD) 10.2 (0.9) versus 9.3 (0.9); and, as median [IQR], *Treponema denticola* 3.2 [IQR 0.0–4.0] versus 4.2 [0.0–5.0]; *Tannerella forsythia* 2.1 [0.0–3.6] versus 3.1 [0.0–4.2]; *Fusobacterium nucleatum* 2.8 [0.0–3.5] versus 0.0 [0.0–2.9]; and *Capnocytophaga gingivalis* 3.9 [3.0–4.3] versus 4.1 [3.4–5.0]. Higher levels of two red-complex organisms coincided with a lower total load in the same patients.

### 3.3. Univariable and Multivariable Associations

Deeper pockets (OR 3.29 per mm), higher right IMT (OR 2.07 per 0.1 mm), higher WBC (OR 1.94), CRP and glucose were associated with higher odds of stroke. A greater number of teeth (OR 0.82 per additional tooth), good oral hygiene (OR 0.12 versus poor) and higher total, HDL and LDL cholesterol and ejection fraction were associated with lower odds. Higher total bacterial load was associated with lower odds of stroke (OR 0.27 per ten-fold increase; 95% CI 0.15–0.49). Among taxa, only *Tannerella forsythia* reached significance in the expected positive direction (OR 1.24), and *Fusobacterium nucleatum* was inversely associated (OR 0.76). The final model retained age, number of teeth, WBC, right IMT, sex and total bacterial load; left IMT was removed during selection. Lower total bacterial load remained independently associated with stroke (adjusted OR 0.09 per ten-fold increase; 95% CI 0.02–0.38; *p* = 0.001; Table 3; Figure 1G). Discrimination was high (c-statistic 0.970; 95% CI 0.932–0.995; McFadden R^2^ 0.694; Nagelkerke R^2^ 0.824; Hosmer–Lemeshow *p* = 0.83), with all variance-inflation factors below 2.2. Stroke severity did not account for the oral or microbiological findings. Admission NIHSS score was unrelated to total bacterial load (Spearman rho −0.04, *p* = 0.78), number of teeth (rho −0.14, *p* = 0.34), oral hygiene grade (rho −0.13, *p* = 0.38) and pocket depth (rho 0.04, *p* = 0.76), and admission mRS score behaved similarly. The only association approaching significance was between discharge mRS score and number of teeth (rho −0.27, *p* = 0.06).

### 3.4. The Inverse Association Is Not Explained by Tooth Loss

Total bacterial load was uncorrelated with tooth number (rho 0.12; *p* = 0.26; Figure 1F). The inverse association persisted after normalisation per tooth (OR 0.40 per ten-fold increase; 95% CI 0.24–0.67) and after adjustment for tooth number (OR 0.16; 95% CI 0.06–0.39). The lower burden in cases is therefore independent of dentition.

### 3.5. Model Discrimination Reflects Case–Control Separability

The apparent c-statistic of 0.970 is higher than is realistic for prospective prediction. An optimism-corrected bootstrap estimated modest optimism (0.030), giving 0.940, indicating that the value is not driven mainly by overfitting but by case–control structure, in which groups differ along several axes at once. Total bacterial load alone yielded a c-statistic of 0.78, and the addition of teeth, age and WBC contributed further group-separating information. In a prospective cohort, the same model would be expected to discriminate less well (Figure 1H).

## 4. Discussion

The analysis yielded a consistent set of observations. The expected clinical picture was reproduced, with cases showing worse periodontal status, greater carotid IMT and higher systemic inflammation than controls, consistent with prior work linking periodontal disease to cerebrovascular disease [6,7]. The subgingival microbiome differed in composition rather than uniformly, with *Treponema denticola* and *Tannerella forsythia* higher in cases and *Fusobacterium nucleatum* lower. Total bacterial load, contrary to the dominant paradigm, was lower in cases, an association independent of measured confounders and unexplained by tooth loss.

The clinical findings are concordant with prior work. Higher carotid IMT in cases is consistent with its role as a cerebrovascular-risk marker and its link to periodontitis [8,9], while higher WBC and CRP are expected after an acute event and do not establish an antecedent inflammatory state. This concordance indicates that the reduced bacterial load in cases did not arise from an unrepresentative sample.

The taxon findings are compatible with existing biology. Higher *Treponema denticola* and *Tannerella forsythia* fit the red-complex association and the keystone model, in which specific organisms rather than numbers drive pathology [1]. Lower *Fusobacterium nucleatum* indicates a directional, species-specific difference, in agreement with sequencing studies that report compositional rather than monotonic change [11,12] and with thrombus work enriched for *Streptococcus* spp. and *Prevotella* spp. but not classical periodontal pathogens [13].

These differences are better read as a change in composition than as a change in quantity. *Treponema denticola* and *Tannerella forsythia* were higher in cases while the total load was lower, so the proportion of the community accounted for by red-complex organisms rose even as the community itself contracted. *Fusobacterium nucleatum*, an early coloniser that bridges the biofilm and is not itself a red-complex organism, moved in the opposite direction. *Porphyromonas gingivalis* did not differ between groups (*p* = 0.804), which is consistent with its keystone role: it exerts its effect at low abundance and need not be numerically dominant to shape the community around it. Taken together, the subgingival profile in manifest stroke is not a scaled-down version of the control profile but a differently constituted one, and it is the composition rather than the summed load that carries the biological signal.

The lower total bacterial load in cases runs opposite to the direction reported for subclinical atherosclerosis in stroke-free cohorts [8,9] and to serological data in which *Porphyromonas gingivalis* exposure predicted incident stroke [10]. This apparent conflict is resolved by considering when and in whom burden was assessed. Those studies sampled exposure before any event, in stroke-free people, and described a forward-directed relationship, whereas the present measurement was made after the stroke, in hospitalised patients.

Several peri-stroke processes plausibly reduce the measured oral load in cases. Antibiotics administered around admission reduce bacterial counts directly, and a prospective study documented an antibiotic-attributed shift in the oral microbiota over the days after admission [17]. Serial sampling after stroke has likewise shown that the oral microbial community shifts and then partially recovers over the days following admission [18]. Dysphagia, common after stroke and frequently managed by restriction of oral intake, together with interrupted hygiene, reduced salivary flow and acute illness, further alters the subgingival niche [17]. Habitual oral hygiene also shapes the pocket community independently of disease status: in neurosurgical patients, more frequent toothbrushing was associated with lower bacterial diversity in gingival crevicular fluid [19]. Any change in oral care around admission can therefore be expected to move both the quantity and the composition of the subgingival community. Consistent with a post-event interpretation, controls carried a higher load than cases despite cases having the higher carotid IMT, a pattern that a forward-causal model does not accommodate. Cross-sectional load in stroke patients is therefore better understood as reflecting the peri-acute state than antecedent exposure.

This interpretation also reconciles the taxon and total-load results. Composition can shift toward particular pathogens while the overall count falls, if the peri-stroke environment suppresses load unevenly across taxa. Selective persistence of red-complex organisms against a lower background would produce the observed pattern, and the two findings are therefore complementary facets of one process rather than conflicting signals.

Two implications follow. Bacterial load measured after the event should not be interpreted as antecedent exposure or entered into risk models as such; the near-0.97 c-statistic reflects case–control separability rather than prospective skill and would be expected to fall substantially in external application. The taxon-specific signal, being consistent with mechanism, merits prospective study that samples the microbiome before the event, records peri-stroke antibiotic exposure and onset-to-sampling interval, and follows participants forward, so that pre-existing exposure can be distinguished from peri-event change.

A clinical caveat follows from the direction of this result. Because total bacterial load was lower in cases, the finding could be misread as indicating that subgingival bacteria are protective, or that oral hygiene matters little for cerebrovascular risk. Neither inference is supported. The present design tested no intervention and sampled the microbiome after the event, so it cannot address prevention. The evidence that does addfigureress prevention is prospective and population-based: dental prophylaxis, regular scaling and regular dental attendance are each associated with lower stroke incidence, with dose–response gradients across increasing frequency of care [20,21,22], as synthesised for stroke specifically elsewhere [23]. A healthy mouth, healthy periodontal tissues, sustained oral hygiene and regular dental review therefore remain a reasonable component of comprehensive cardiovascular and stroke prevention. The present findings constrain how bacterial load measured after an event should be interpreted; they do not weaken the case for oral health maintenance.

### Limitations

The study has design features that support internal comparison, including concurrent recruitment to both arms, joint quantification of nine named pathogens and total load, and concurrent carotid imaging. Several limitations qualify the findings. This cross-sectional comparison quantifies associations with prevalent stroke and cannot establish causation. Microbial and inflammatory markers were measured after the event and may be consequences rather than antecedents. The sample is modest and single-centre, with men over-represented among cases. Ischaemic stroke was not classified by aetiological subtype according to a formal system such as TOAST. Imaging identified a lacunar infarct in 6 of 50 cases and a territorial infarct in 40, but this does not establish mechanism, and the case group necessarily includes infarcts that are not primarily atherosclerotic; atrial fibrillation was documented in only three participants overall, too few to support any subtype-specific analysis. Backward elimination means confidence intervals and *p* values are optimistic, and taxon comparisons were not corrected for multiple testing, so single-taxon associations are hypothesis-generating. Finally, none of these constraints is removed by a more elaborate analytical approach. Automated and machine-learning methods can accelerate the analysis of routinely collected clinical data, including an algorithm previously developed by our group for audit and quality improvement [24], but they inherit the temporal structure of the data supplied to them: a model fitted to measurements made after the event will reproduce the same non-causal separation, however it is estimated. Echocardiography and ambulatory electrocardiography were performed as part of routine clinical care, so the equipment and the measurement protocol were not standardised for this study; ejection fraction should therefore be read as a clinical estimate rather than a study measurement. The microbiological analysis was performed with a commercial assay whose detection threshold, calibration and internal controls are proprietary, so these could not be reported; counts below the threshold are returned as non-detections and were analysed as zero, which will understate the load of taxa present at very low abundance.

## 5. Conclusions

In manifest ischaemic stroke, the subgingival microbiome shifted in a taxon-specific manner, but total bacterial load was lower, not higher, than in stroke-free controls, independent of tooth loss. These findings do not overturn the periodontal-burden paradigm; they define its boundary. Burden measured after the event behaves differently from exposure assessed in stroke-free cohorts, most plausibly through peri-event antibiotics and disruption of the oral environment. Prospective sampling, explicit capture of antibiotic exposure, and external validation are required before oral bacterial load could enter cerebrovascular risk assessment.

## Figures and Tables

**Table 1:** Baseline characteristics by group. Atrial fibrillation status was available for 95 participants (48 controls, 47 cases) and percentages are based on those denominators. Values are mean (SD) or median [IQR] by distribution; categorical variables *n* (%). Selected variables are shown; HDL cholesterol and left carotid intima-media thickness were measured and are reported in the text but are not tabulated here.

Characteristic	Overall (*N* = 98)	Control (*n* = 48)	Stroke (*n* = 50)	*p*
Age, y	66.7 (8.2)	67.4 (7.0)	66.0 (9.1)	0.377
Male sex, *n* (%)	58 (59.2)	21 (43.8)	37 (74.0)	0.005
Current smoking, *n* (%)	53 (54.1)	23 (47.9)	30 (60.0)	0.319
No. of teeth	24.5 [16.0–28.0]	28.0 [25.0–30.0]	18.0 [11.2–24.0]	<0.001
Pocket depth, mm	3.0 [2.0–3.0]	2.0 [2.0–3.0]	3.0 [3.0–3.0]	<0.001
Good oral hygiene, *n* (%)	48 (49.0)	34 (70.8)	14 (28.0)	<0.001
Carotid IMT, right, mm	0.8 (0.2)	0.7 (0.1)	0.9 (0.2)	<0.001
WBC, ×10^3^/µL	7.1 [5.8–9.1]	6.2 [5.3–7.1]	8.7 [7.5–10.7]	<0.001
CRP, mg/dL	0.7 [0.1–3.5]	0.1 [0.0–0.2]	3.2 [1.0–8.4]	<0.001
Glucose, mg/dL	105.5 [94–120]	101 [92–109]	112.5 [102–145]	<0.001
Total cholesterol, mg/dL	193 [156–241]	210 [175–246]	173 [139–224]	0.010
LDL cholesterol, mg/dL	117.2 (45.6)	127.9 (42.5)	106.9 (46.5)	0.022
Ejection fraction, %	65 [60–65]	65 [65–65]	60 [58–63]	<0.001
Atrial fibrillation, *n* (%)	3 (3.2)	1 (2.1)	2 (4.3)	0.617

**Table 2:** Subgingival bacterial counts by group, on the log10 scale. The total bacterial count is given as mean (SD) and individual taxa as median [interquartile range], following the distribution of each variable; groups were compared with the Student *t*-test or the Mann–Whitney U test accordingly. Individual taxa were analysed as log10(count + 1) so that zero counts remain defined. Numbers analysed range from 92 to 97 depending on missing samples.

Taxon (log10 Scale)	Control (*n* = 48)	Stroke (*n* = 50)	*p*-Value
Total bacterial count	10.2 (0.9)	9.3 (0.9)	<0.001
*Porphyromonas gingivalis*	2.0 [0.0–4.4]	0.0 [0.0–4.9]	0.804
*Treponema denticola*	3.2 [0.0–4.0]	4.2 [0.0–5.0]	0.013
*Tannerella forsythia*	2.1 [0.0–3.6]	3.1 [0.0–4.2]	0.047
*Prevotella intermedia*	0.0 [0.0–0.0]	0.0 [0.0–3.7]	0.107
*Peptostreptococcus* (*Micromonas*) *micros*	2.9 [2.2–3.4]	3.0 [0.0–3.7]	0.741
*Fusobacterium nucleatum*	2.8 [0.0–3.5]	0.0 [0.0–2.9]	0.020
*Capnocytophaga gingivalis*	3.9 [3.0–4.3]	4.1 [3.4–5.0]	0.049
*Aggregatibacter actinomycetemcomitans*	0.0 [0.0–0.0]	0.0 [0.0–0.0]	0.525
*Eubacterium nodatum*	0.0 [0.0–0.0]	0.0 [0.0–0.0]	0.498

**Table 3:** Multivariable logistic model for ischaemic stroke (adjusted odds ratios); 89 complete cases, 43 events.

Variable	Adjusted OR (95% CI)	*p*
Age, per year	0.84 (0.73–0.96)	0.014
Female sex	0.19 (0.03–1.24)	0.082
No. of teeth, per tooth	0.75 (0.62–0.89)	0.001
Carotid IMT, right, per 0.1 mm	1.68 (0.93–3.02)	0.083
WBC, per ×10^3^/µL	1.60 (1.06–2.40)	0.025
Total bacterial load, per 10-fold	0.09 (0.02–0.38)	0.001

## Data Availability

The data presented in this study are available on request from the corresponding author due to privacy restrictions relating to individual patient data.
